# Human prion protein-induced autophagy flux governs neuron cell damage in primary neuron cells

**DOI:** 10.18632/oncotarget.8802

**Published:** 2016-04-19

**Authors:** Ji-Hong Moon, Ju-Hee Lee, Uddin MD Nazim, You-Jin Lee, Jae-Won Seol, Seong-Kug Eo, John-hwa Lee, Sang-Youel Park

**Affiliations:** ^1^ Biosafety Research Institute, College of Veterinary Medicine, Chonbuk National University, Iksan, Jeonbuk, South Korea

**Keywords:** prion protein, autophagy flux, neurodegeneration, PrPc, ATG5, Gerotarget

## Abstract

An unusual molecular structure of the prion protein, PrPsc is found only in mammals with transmissible prion diseases. Prion protein stands for either the infectious pathogen itself or a main component of it. Recent studies suggest that autophagy is one of the major functions that keep cells alive and has a protective effect against the neurodegeneration. In this study, we investigated that the effect of human prion protein on autophagy-lysosomal system of primary neuronal cells. The treatment of human prion protein induced primary neuron cell death and decreased both LC3-II and p62 protein amount indicating autophagy flux activation. Electron microscope pictures confirmed the autophagic flux activation in neuron cells treated with prion protein. Inhibition of autophagy flux using pharmacological and genetic tools prevented neuron cell death induced by human prion protein. Autophagy flux induced by prion protein is more activated in prpc expressing cells than in prpc silencing cells. These data demonstrated that prion protein-induced autophagy flux is involved in neuron cell death in prion disease and suggest that autophagy flux might play a critical role in neurodegenerative diseases including prion disease.

## INTRODUCTION

The impilation of protein aggregates in affected brains is associated with pathogenesis of most neurodegenerative diseases including Alzheimer's disease and Parkinson's disease, as well as prion diseases [1]. Though the incidence of prion diseases is rare, it is lethal because of a lack of efficient treatment to either cure or delay disease progression. Moreover, the risk of prion transmission is a severe threat to public health due to the high resistance of prion components to standard sterilization techniques [2]. A wealth of research supports the contention that scrapie prions are devoid of nucleic acid and seem to be composed exclusively of a modified isoform of PrP, designated PrPsc [3]. The normal cellular PrP, meaning PrPc, is converted into PrPsc through a process whereby a section of its α-helical and coil construction is refolded into β sheet [4]. These profound changes in the physicochemical properties of the PrP bring out structural transition. While PrPc is soluble in nondenaturing detergents, PrPsc is not. PrPc is smoothly digested by proteases, whereas PrPsc is resistant in part [5]. It is still unknown whether scrapie pathology takes effect by neurotoxicity of PrPsc, severe depletion of PrPc, or some other mechanism [6].

A synthetic peptide corresponding to amino acid residues 106-126 of human PrP which forms fibrils *in vitro* has previously been demonstrated to be toxic to cultured hippocampal neurons [7]. It may be hypothesized that a toxic form of PrP is produced directly from PrPc or as a precursor to pathological PrP [8]. The significant fact was that *Prnp*^−/−^ mice are resistant to infection with prion agents [9–11]. Secondly, *Prnp*^−/−^ primary culture neurons are not executed by the toxicity of prion agents or PrP (106-126) [6, 12].

Prion diseases are associated with misregulation of autophagy as shown by the formation of giant autophagic vacuoles in experimental scrapie in hamsters [13]. These autophagic vacuoles often grow in size and number as neurons age, eventually occupying the entire volume of the correlated neurites [14].

Autophagy, called programmed cell death II, is the generic term employed for any intracellular process that results in the degradation of cytosolic ingredients inside lysosomes [15]. Programmed cell death is especially relevant in the remodeling of the developing heart and in several pathological conditions such as hypoxia, ischemia, inflammation and exposure to toxic agents [16]. The participation of the lysosomal system in programmed cell death is well accepted recently, though its specific role is still controversial [17]. By eliminating intracellular damaged organelles and aggregates, autophagy promotes cell surface antigen presentation and cellular senescence, protects against genome instability and prevents necrosis, offering it an essential role in preventing diseases such as neurodegeneration, cancer, cardiomyopathy, diabetes, liver disease, autoimmune diseases and infections [18].

A recent study looking at the degradation of PrPsc has demonstrated the crucial role of the lysosome in prion degradation and autophagy appears to be the main route of PrPsc delivery to lysosomes [19–21]. The authors proposed autophagy flux is a therapeutic mechanism in several diseases. Xu *et al*. insisted that the macro-autophagic system was activated in scrapie-infected experimental animals and human prion diseases [22]. On the basis of the results of many studies, we expected that prion peptide treatment induced autophagy activation or impairment in neuronal cells. Briefly, we investigated whether prion peptide induced autophagy flux or autophagy flux impairment to identify that autophagy flux is apoptotic or a protective pathway. We determined PrP (106-126) induced autophagy flux dose-dependently in neuronal cells. In this study, we examined the effect of prion peptide-induced autophagy in neuronal cells. The data will support the hypothesis that autophagy inhibition may be a therapeutic implication for prion diseases.

## RESULTS

### Prion peptide induced autophagy flux and neurotoxicity in neuronal cells

**Figure 1:**
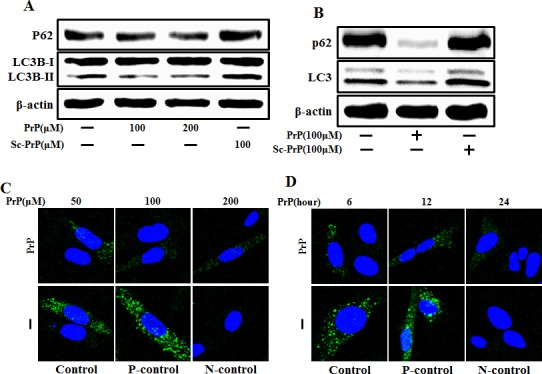

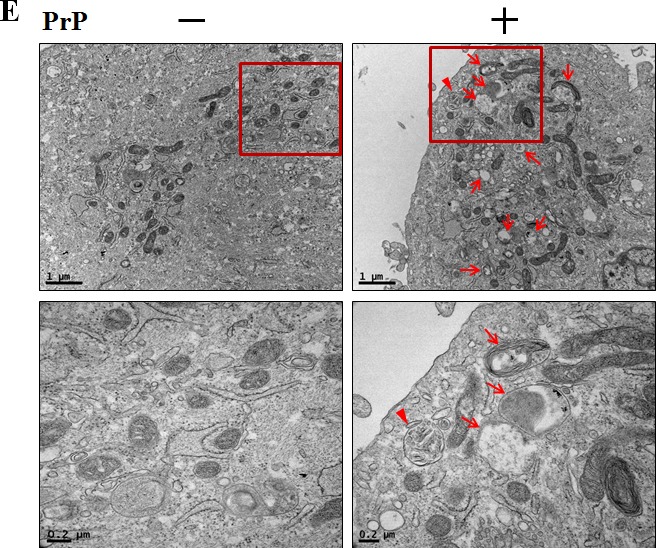
PrP (106-126) treatment induced autophagy flux **A.** SK-N-SH neuroblastoma cells were treated with PrP (106-126) in a dose dependent manner or scrambled PrP for 6h. **B.** The primary neuron cells were treated with PrP (106-126) or scrambled PrP for 6h. The treated cells were assessed for LC3B production and P62 expression by western blot analysis. SK-N-SH cells were mixed with a titration (30MOI) of BacMam GFP-LC3B virus over 18h, and then treated with PrP (106-126) in a dose-dependent manner**C.** for 6h and time-dependent manner **D.**, Negative control reagent and positive control reagent (CQ) at the same time. SK-N-SH cells were stained with DAPI (nuclei, blue) and analyzed by confocal microscopy. **E.** SK-N-SH cells were incubated with PrP (106-126) at 100μM for 6 h and analyzed by TEM. Arrowheads indicate autophagosomes and arrows indicate autolysosomes. PrP, Prion peptide (106-126); sc-PrP, scrambled Prion peptide; P-control, Positive control; N-control, Negative control.

To determine the activity of the autophagic system during prion pathogenesis, the alterations of the SQSTM1/p62 protein, which serves as a link between LC3 and ubiquitinated substrates and is able to be incorporated into, and then degraded by autophagosomes [23], were evaluated in mouse primary neuronal cells. We investigated prion peptide induced autophagy flux by estimating LC3B transformation and SQSTM1/p62 expression. LC3 protein is localized and aggregated on autophagosome and is therefore considered to be a marker of autophagy. LC3 transforms from LC3-I to LC3-II during autophagosome formation [24]. As shown in Figure 1, we identified levels of the late autophagosome marker LC3-II and SQSTM1/p62 decreased in the prion peptide-treated group in a dose-dependent manner compared with the control group through western blot analysis in human neuroblastoma cells and mouse primary neuron cells (Figure 1A, 1B). To visualize in neuronal cells the activation of the autophagy process through the formation of autophagosomes, the Premo Autophagy Sensor (LC3B-FP) BacMam 2.0 system was employed. According to the results reported in Figure 1C and 1D, SK-N-SH cells treated with PrP (106-126), presented a reduced punctate fluorescent distribution pattern, suggesting LC3B-FP protein accumulation in autophagosomes. We analysed this decrease of LC3-II and green fluorescent puncta was caused by lysosomal degradation of autophagosomes. For further detection of autophagic flux, transmission electron microscopy was performed. As shown in Figure 1E, single-membraned autolysosomes, containing entrapped cytoplasm or entire organelles, were induced by PrP (106-126) treatment. Thus, these results suggest that prion peptide activates autophagy flux in human and mice neuronal cells.

**Figure 2:**
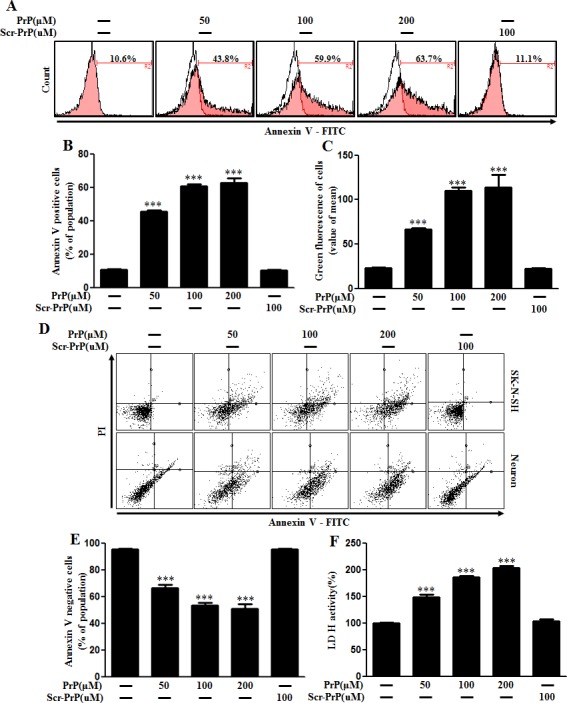
PrP (106-126) treatment induced neuronal cytotoxicity **A.** Primary neuronal cells were treated with PrP (106-126) in a dose-dependent manner for 24h. Cell viability was measured by annexin V assay. Cells were treated with FITC-annexin V, which binds to phosphatidylserine to the plasma membrane during apoptosis. **B.** Bar graph indicating the average number of annexin V positive cells. **C.** Bar graph displaying the mean value of green fluorescence of total cells. **D.** Primary neuronal cells and SK-N-SH neuroblastoma cells were treated with PrP (106-126) in a dose-dependent manner for 24h. Cell viability was measured by annexin V assay. Cells were treated with FITC-annexin V and PI, which binds to phosphatidylserine to the plasma membrane and nuclei during apoptosis. **E.** Bar graph indicating the average number of annexin V negative cells. **F.** Lactate dehydrogenase (LDH) assay was used to quantify LDH released into the medium. *** *p* < 0.001; significant differences between each treatment group. PrP, Prion peptide (106-126); sc-PrP, scrambled Prion peptide.

SK-N-SH neuroblastoma cells were exposed to PrP (106-126) for 24 hr dose-dependently. When neuronal cells were treated with PrP (106-126), it resulted in neurotoxicity as measured by Annexin V (Figure 2A, 2B, and 2C). We also confirmed neurotoxicity was induced by PrP (106-126) in mice primary neuronal cells as demonstrated by Annexin V and PI staining (Figure 2D, 2E). We measured cell viability using scrambled PrP (106-126) as a control condition for PrP 106-126. As a result, cell death caused by scrambled PrP (106-126) did not occur. LDH release levels showed that prion peptide induced apoptosis in a dose-dependent manner but scrambled PrP did not lead to cell death (Figure 2F). These results suggest that prion peptide (106-126) activates autophagic flux and neuronal apoptosis in mammalian cells.

### Inhibition of autophagy flux alleviated prion protein-induced neurotoxicity

We recognized that the specific role of autophagy flux is still controversial. Therefore we set out to determine if autophagy flux has a protective function or not.

**Figure 3:**
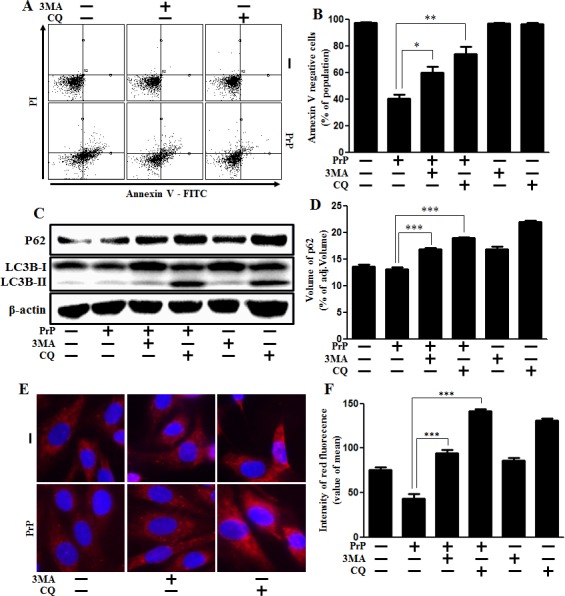

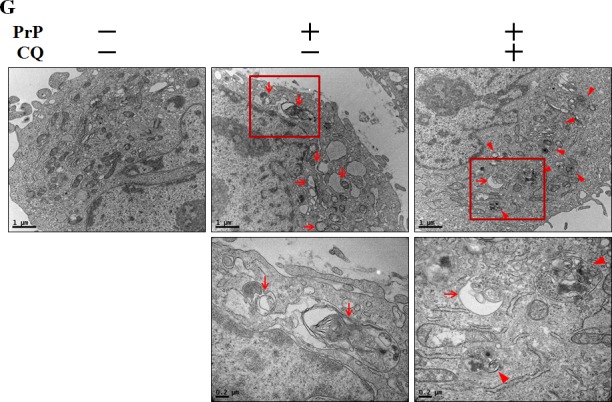
Autophagy inhibition alleviated PrP (106-126)-induced cytotoxicity **A.** SK-N-SH neuronal cells were pretreated with autophagy inhibitors (3MA, chloroquine) (1h) and then exposed to PrP (106-126) with 100μM for 24h. Cell viability was measured by annexin V assay. Cells were treated with FITC-annexin V and PI, which binds to phosphatidylserine to the plasma membrane and nuclei during apoptosis. **B.** Bar graph indicating the average number of annexin V negative cells. **C.** Primary neuron cells were pretreated with autophagy inhibitors (3MA, chloroquine) (1h) and then exposed to PrP (106-126) with 100μM for 6h. The treated cells were assessed for LC3B production and P62 expression by western blot analysis. β-actin was used as loading control. **D.** Bar graph indicating the average values of p62 expression levels. **E.** SK-N-SH cells were stained with rabbit anti-p62 (red) and DAPI (nuclei, blue) for immunocytochemistry using confocal microscopy. **F.** Bar graph displaying the intensity of red fluorescence (p62). **G.** SK-N-SH cells were pre-incubated with chloroquine (1h) and then exposed to PrP (106-126) at 100μM for 6 h and analyzed by TEM. Arrowheads indicate autophagosomes and arrows indicate autolysosomes. * *p* < 0.05, ** *p* < 0.01,*** *p* < 0.001; significant differences between each treatment group. PrP, Prion peptide (106-126); CQ, chloroquine; adj.volume, adjustment of volume (band volume minus background volume).

Firstly, we confirmed the effects of 3MA and CQ on prion peptide-induced neurotoxicity in neuronal cells. We demonstrated that 3MA and CQ enhanced cell viability decreased with prion peptide treatment (Figure 3A, 3B). We also examined whether autophagy inhibition was conducted by autophagy inhibitors (3MA, chloroquine (CQ)) using western blot analysis (Figure 3C). We confirmed that prion peptide-induced autophagy flux was inhibited by 3MA and CQ by identifying up-regulation of SQSTM1/p62 protein (Figure 3D). These results were also supported by additional experimental data using immunocytochemistry by confocal microscope (Figure 3E). We also analyzed intensity of fluorescence using graph (Figure 3F). To certainly determine the effect of lysosomal inhibition on autophagy flux by chloroquine, transmission electron microscopy was implemented. As shown in Figure 3G, a lot of vesicles including double-membraned autophagosomes (arrowheads) were induced by treatment of cells with chloroquine, which indicated inhibition of lysosomal degradation.

**Figure 4:**
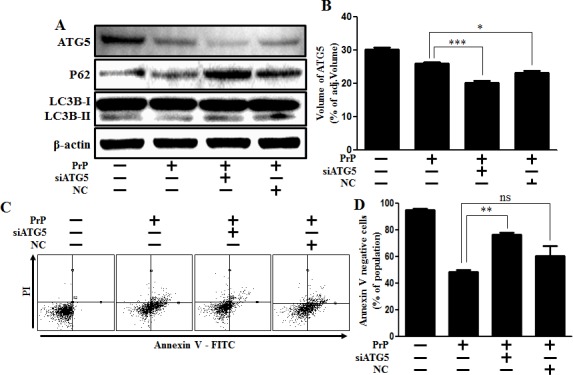
Inhibition of ATG5 gene expression alleviated PrP (106-126)-induced cytotoxicity **A.** ATG5 small interfering RNA (siATG5) or negative control siRNA (NC) transfected SK-N-SH neuronal cells were incubated with 100 μM PrP (106-126) for 6h. Western blot for LC3-II and p62 proteins was analyzed from SK-N-SH cells. Beta-actin was used as the loading control. **B.** Bar graph indicating the volume of ATG5 expression levels. **C.** Cell viability was measured by annexin V assay. siATG5 or NC transfected SK-N-SH neuron cells were incubated with 100 μM PrP (106-126) for 24h. **D.** Bar graph indicating the average number of annexin V negative cells. * *p* < 0.05, ** *p* < 0.01, *** *p* < 0.001; significant differences between each treatment group. PrP, Prion peptide (106-126); adj.volume, adjustment of volume (band volume minus background volume).

We further tested whether autophagy inhibition by knockdown of gene levels could decrease prion peptide-induced neurotoxicity. Knockdown of ATG5 using ATG5 small interfering RNA (ATG5 siRNA) inhibited prion peptide-induced autophagy flux (Figure 4A, 4B), as well as attenuated the neurotoxicity caused by prion peptide treatment in SK-N-SH neuronal cells (Figure 4C, 4D). Our results show that autophagy inhibition has a protective influence on prion peptide-induced neurotoxicity.

### Autophagy induction enhanced prion peptide-induced neuronal apoptosis

**Figure 5:**
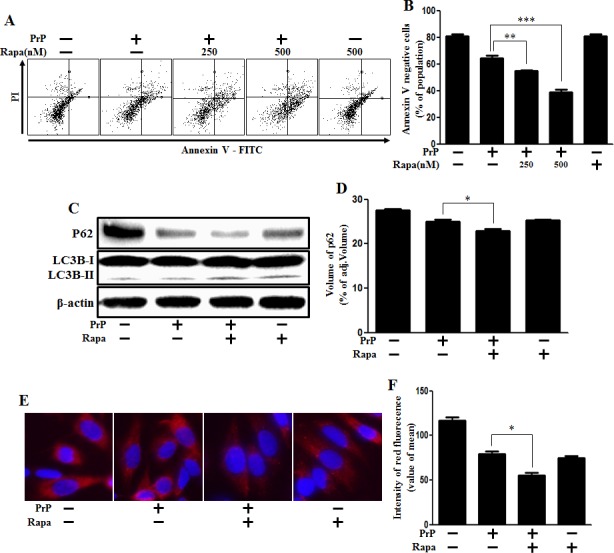
Rapamycin enhanced PrP (106-126)-induced Autophagy and cytotoxicity **A.** Primary neuronal cells were pretreated with autophagy inducer (rapamycin) (18h) and then exposed to PrP (106-126) with 100μM for 24h. Cell viability was measured by annexin V assay. Cells were treated with FITC-annexin V and PI, which binds to phosphatidylserine to the plasma membrane and nuclei during apoptosis. **B.** Bar graph indicating the average number of annexin V negative cells. **C.** Primary neuron cells were pretreated with autophagy inducer (rapamycin) (18h) and then exposed to PrP (106-126) with 100μM for 6h. The treated cells were assessed for LC3B production and P62 expression by western blot analysis. β-actin was used as loading control. **D.** Bar graph indicating the average levels of p62 expression levels. **E.** SK-N-SH cells were stained with rabbit anti-p62 (red) and DAPI (nuclei, blue) for immunocytochemistry using confocal microscopy. **F.** Bar graph displaying the intensity of red fluorescence (p62). * *p* < 0.05, ** *p* < 0.01, *** *p* < 0.001; significant differences between each treatment group. PrP, Prion peptide (106-126); Rapa, Rapamycin; adj.volume, adjustment of volume (band volume minus background volume).

Next, we investigated whether autophagy induction could enhance prion peptide-induced neuronal apoptosis. We conducted cell viability tests to investigate whether autophagy induction could enhance prion peptide-induced neuronal apoptosis through rapamycin treatment. Our results show that rapamycin treatment enhanced neuronal apoptosis caused by prion peptide treatment (Figure 5A, 5B). The graph indicated population of dot plot in lower left quadrant that means Annexin V negative cells. We used western blot analysis to identify the function of rapamycin (Figure 5C). We confirmed that prion peptide-induced autophagy flux was enhanced by rapamycin treatment through identifying the reduction of SQSTM1/p62 protein (Figure 5D). These results were also supported by additional experimental data using immunocytochemistry (Figure 5E, 5F).

### Autophagy inhibition by PrPc deficiency protected against PrP (106-126)-induced neurotoxicity

**Figure 6:**
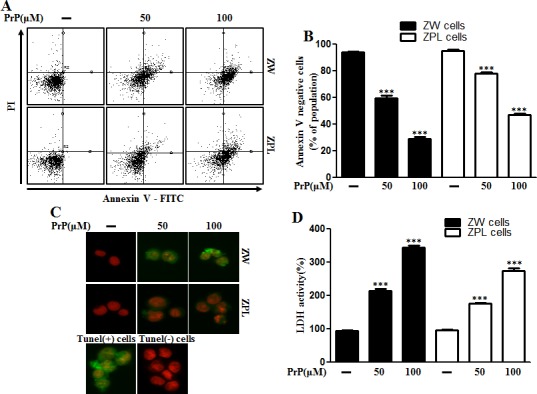

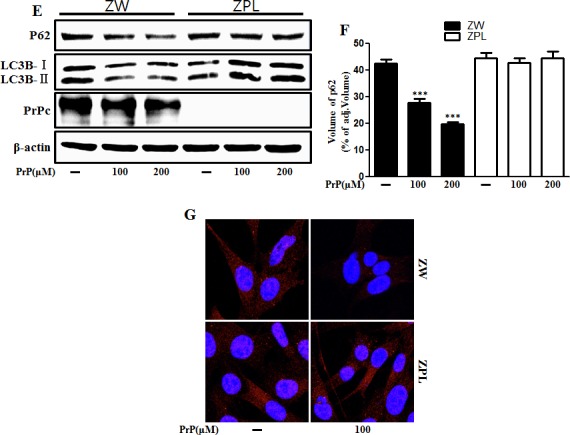
Autophagy and neurotoxicity by PrP (106-126) treatment was inhibited in PrnP deficient cells **A.** ZW 13-2 and Zpl 3-4 cells were incubated with PrP (106-126) in a dose-dependent manner for 24h. Cell viability was measured by annexin V assay. Cells were treated with FITC-annexin V and PI, which binds to phosphatidylserine to the plasma membrane and nuclei during apoptosis. **B.** Bar graph indicating the average number of annexin V negative cells. **C.** Representative immunofluorescence images of TUNEL-positive (green) ZW or Zpl cells at 24 h after exposure to 100 μM of PrP (106-126). The cells were counterstained with PI (red) to show all cell nuclei. **D.** Lactate dehydrogenase (LDH) assay was used to quantify LDH released into the medium. **E.** ZW 13-2 and Zpl 3-4 cells were incubated with PrP (106-126) in a dose-dependent manner for 6h. The incubated cells were assessed for LC3B production, prion protein and P62 expression by western blot analysis. β-actin was used as loading control. Bar graph indicating the average values of p62 expression levels **F.**. **G.** ZW 13-2 and Zpl 3-4 cells were stained with rabbit anti-p62 (red) and DAPI (nuclei, blue) for immunocytochemistry using confocal microscopy. *** *p* < 0.001; significant differences between each treatment group. PrP, Prion peptide (106-126); PrPc, normal prion protein; adj.volume, adjustment of volume (band volume minus background volume).

**Figure 7:**
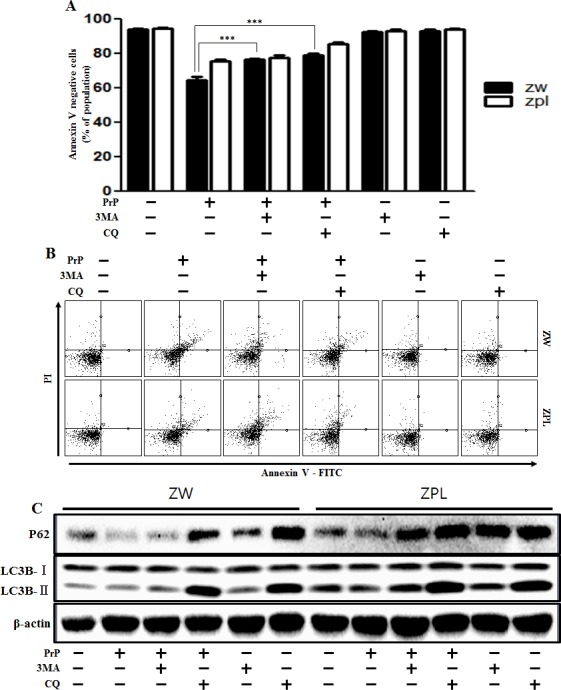
Autophagy flux induced by PrP (106-126) resulted in more neurotoxicity in ZW 13-2 cells ZW 13-2 and Zpl 3-4 cells were pretreated with autophagy inhibitors (3MA, chloroquine) (1h) and then exposed to PrP (106-126) with 100μM for 24h. Cell viability was measured by annexin V assay. Cells were treated with FITC-annexin V and PI, which binds to phosphatidylserine to the plasma membrane and nuclei during apoptosis. Bar graph indicating the average number of annexin V negative cells **A.**, **B.**. **C.** ZW 13-2 and Zpl 3-4 cells were pretreated with autophagy inhibitors (3MA, chloroquine) (1h) and then exposed to PrP (106-126) with 100μM for 6h. The incubated cells were assessed for LC3B production and P62 expression by western blot analysis. β-actin was used as loading control. *** *p* < 0.001; significant differences between each treatment group. PrP, Prion peptide (106-126).

Some studies have examined the relationship between autophagy and prion protein (PrPc) in neurodegeneration [25, 26]. To verify that prion protein deficiency plays a protective role in prion peptide-induced neurotoxic condition, the mouse neuronal cell lines ZW 13-2 and Zpl 3-4 established from the hippocampus of ICR (*Prnp*^+/+^) and Zürich I *Prnp*^−/−^mice, respectively, were cultured. We showed that prion peptide-induced cell death under normal conditions, but PrPc deficiency attenuated prion peptide-induced cell apoptosis in PrPc knockout Zpl 3-4 cells (Figure 6A, 6B). These data indicate that PrPc plays a harmful role in neuron cells. As shown in Figure 6C, prion peptide-induced apoptosis process emitted more green fluorescence (an indication of DNA strand breakage) in ZW 13-2 than Zpl 3-4 cells. LDH release levels also increased more in ZW 13-2 cells (Figure 6D). Western blot assays showed that PrP (106-126) treatment inhibited autophagy flux in Zpl 3-4 cells (Figure 6E, 6F), and that PrPc expression was displayed in ZW 13-2 cells (Figure 6E). In contrast, no detectable PrPc was evident in Zpl 3-4 cells (Figure 6E). We used immunocytochemistry to identify the inhibition of autophagy flux in Zpl 3-4 cells (Figure 6G). In addition, we identified whether autophagy inhibitor (3MA, chloroquine) has protective role against prion peptide neurotoxicity in ZW 13-2 and Zpl 3-4 cells. As shown in Figure 7A and 7B, autophagy inhibitor enhanced cell viability in ZW 13-2 more than Zpl 3-4 cells. We checked autophagy inhibition by autophagy inhibitors (3MA, chloroquine) *via* western blot analysis (Figure 7C). On the basis of the above results, we suggest that prion protein existence has a harmful function and activates apoptotic autophagy flux in prion peptide-induced neurotoxic conditions.

## DISCUSSION

The purpose of this study was to investigate the role of prion peptide-induced autophagy and the regulation of PrP (106-126)-induced apoptosis by autophagy inhibition in neuronal cells.

The results suggest that the up-regulation in autophagy induced by prion peptide and the resultant reduction in PrP (106-126)-induced neurotoxicity by autophagy inhibition might be a key mechanism underlying the observed autophagy flux in prion disease.

Autophagy is an evolutionary dynamic and lysosome-mediated process that entails the sequestration and delivery of cytoplasmic material to the lysosome where it is degraded and recycled [27, 28]. Many studies have proposed that certain chemical properties protect neurons during autophagy in neurodegenerative disease and autophagy is important for the survival and homeostasis in neurons [29–33]. Autophagy has several pathways depending on the circumstances and the biochemical basis for the diverse functions is not well understood [17, 34]. One of the principal methods in current use to measure autophagic flux is the monitoring of LC3 turnover, which is based on the observation that LC3-II is degraded in autolysosomes. Our results indicated that LC3-II was degraded by prion peptide treatment. Mizushima *et al*. suggested that LC3-II, which increases transiently upon induction of autophagy, is decreased after longer periods of autophagy activation [35]. For this reason, we suggest that degradation of LC3 and p62 indicates induction of autophagic flux.

Lee *et al*. suggested that pharmacological autophagy alleviated prion peptide-induced neuronal apoptosis [31, 36, 37]. In contrast, some studies suggested prion is caused by amyloid plaque formed PrPsc aggregates in the brain similar to amyloid beta in AD [38, 39]. Jihoon *et al*. suggested that is autophagy induced by amyloid β in neuronal cells [40]. These studies may support our theory that amyloid plaque like PrPsc aggregates activates autophagy in neurons.

Büeler *et al*. and Manson *et al*. suggested that PrPsc was not toxic to the brains of innoculated PrP-knock-out mice in the absence of PrPc [9, 11]. PrPc is well known to be the main factor resulted in PrPsc transformation. Shin *et al*. reported that deficiency of PrPc induced impaired autophagy flux in neurons [41, 42]. On the basis of these suggestions, we also propose that prion peptide induces autophagy-induced neurotoxicity in existence of prion protein. As we know, the specific role of lysosomal system in programmed cell death is still controversial. Further studies are needed to identify the differing functions of autophagy flux.

We suggest that prion peptide may have critical role as a therapeutic strategy for prion diseases though PrP (106-126) is not equivalent to PrPsc. We found the 106-126 sequence of the prion protein is an efficient model for *in vitro* study of prion-induced cell death, as it results in rapid depolarization of mitochondrial membranes. Several studies have shown that PrP (106-126) induces apoptotic cell death *in vivo* in model retinal neurons treated with an intra-vitreous injection of PrP fragments [43]. As we only conducted experiments with prion peptide in cells, not animal models, the autophagic effects of prion peptide have yet to be demonstrated *in vivo*. Further studies are needed to discover whether prion peptide induces the neurotoxic effect that occurs in autophagy pathways in mice models to research potential anti-prion drugs and their therapeutic role in prion diseases.

## MATERIALS AND METHODS

### Cell culture

The primary neurons were isolated from embryonic 18-day mice. Briefly, tissues were collected in Hanks Buffered Saline Solution without Mg2+ and Ca2+ (HBSS: Invitrogen-GIBCO, Grand Island, NY, USA), and digested in 0.25% trypsin with DNAse I (2000 Units/mg) (Invitrogen, Carlsbad, CA, USA) for 20min at 37°C. Cells were mechanically dissociated and diluted in HBSS containing Mg2+ and Ca2+. Isolated neuron cells were diluted in DMEM containing 25mM glucose (abbreviated in text as ‘glucose’ medium; Thermo scientific) supplemented with 10% FBS, and plated in flasks pre-coated with 50μg/ml poly D-lysine. The human neuroblastoma cell line SK-N-SH was obtained from the American Type Culture Collection (ATCC, Rockville, MD, USA). Mouse neuronal cell lines, ZW 13–2 and Zpl 3–4 established from the hippocampus of ICR (*Prnp/*) and Zürich I *Prnp/* mice, respectively, were provided by Professor Yong-Sun Kim (Hallym University, Chuncheon, Kangwon-do, South Korea). Cells were cultured in Minimum Essential Medium (MEM, Hyclone Laboratories, Logan, UT, USA) containing 10% fetal bovine serum (Invitrogen-GIBCO, Grand Island, NY, USA) and gentamycin (0.1 mg/mL) in a humidified incubator maintained at 37°C and 5% CO_2_.

### PrP (106-126) treatment

Synthetic PrP (106-126) peptides (sequence, Lys-Thr-Asn-Met-Lys-His-Met-Ala-Gly-Ala-Ala-Ala-Ala-Gly-Ala-Val-Val-Gly-Gly-Leu-Gly) were synthesized by Peptron (Seoul, Korea). The peptides were dissolved in sterile dimethyl sulfoxide at a stock concentration of 10 mM and stored at −20°C.

### Annexin V assay

Apoptosis in detached cells was assessed using an annexin V Assay kit (Santa Cruz Biotechnology, Santa Cruz, CA, USA) according to the manufacturer's protocol. Annexin V levels were determined by measuring fluorescence at 488-nm excitation and 525/30 emission using a Guava EasyCyte HT System (Millipore, Bedford, MA, USA).

### Terminal deoxynucleotidyl transferase dUTP nick end labeling (TUNEL) assay

TUNEL analysis was performed to measure the degree of cellular apoptosis using an *in situ* ApoBrdU DNA fragmentation assay kit (BioVision, Mountain View, CA, USA), following the manufacturer's instructions. Cells were counterstained with propidium iodide (PI) to show cell nuclei.

### Lactate dehydrogenase assay

Cytotoxicity was assessed in supernatants using a lactate dehydrogenase (LDH) cytotoxicity detection kit (Takara Bio, Inc., Tokyo, Japan) according to the manufacturer's protocol. LDH activity was determined by measuring absorbance at 490 nm, using a microplate reader (Spectra Max M2, Molecular Devices, Sunnyvale, CA, USA).

### BacMam transduction

Wild-type or mutant GFP-tagged LC3B was expressed in cells by adding the appropriate concentrations of appropriate virus from the Premo Autophagy Sensor LC3B-GFP (BacMam 2.0) kit (Life Technologies P36235) to the growth medium. LC3B-FP and LC3B (G120A)-FP viral vectors (MOI = 30) were transduced into cells, enabling the expression of fluorescent LC3B protein, and consequently, monitoring of autophagosomes dynamics using an inverted fluorescent microscope analysis. Negative control was established by using mutant chimera LC3B (G120A)-FP.

### Immunocytochemistry

Immunocytochemical analyses were performed on neuroblastoma cells with anti-p62 (P0067, Sigma Aldrich). Cells were cultured on Slide Glass (Nalge Nunc International, Naperville, IL). Cells were washed in sterilized TBST for 10 min, then blocked for 15 min with 5% FBS in TBST, and then incubated overnight at 4°C with the primary antibodies diluted with 5% FBS in TBST. Alexa Fluor 488-labeled donkey anti-rabbit IgG antibody diluted 1:1000 (Molecular Probes, A21206) was used to visualize channel expression using fluorescence microscopy.

### Confocal microscopy

After treatment, coverslips were fixed with 4%PFA in PBS for 15-20 min at room temperature (RT) and permeabilized in 0.3%Triton-X100 in PBS supplemented with 5% horse serum for 10 min. Subsequent incubations were carried out in the permeabilization buffer. Coverslips were incubated with appropriate primary antibodies for 60 min at RT, washed 4 times in PBS and incubated with AlexaFluor-488, AlexaFluor568- and AlexaFluor647-conjugated secondary antibodies at a concentration of 0.3 μg/ml each for 60 min at RT. Coverslips were then mounted in mounting medium (Southern) and imaged on a Zeiss LSM710 microscope equipped with a standard set of lasers through a 63× oil objective. Excitation wavelengths were 488, 543 nm and 633 nm. Bandpass filters were set at 500-550 (AlexaFluor488), 560-615 nm (Cy3, AlexaFluor568) and 650-750 nm (AlexaFluor647). Image acquisition was carried out at the 12-bit rate. Settings were optimized to ensure appropriate dynamic range, low background and sufficient signal/noise ratio.

### RNA interference

SK-N-SH cells were transfected with ATG5 small interfering RNA (siRNA: oligoID HSS114104: Invitrogen, Carlsbad, CA, USA) or Sirt1 siRNA (oligoID VHS50608: Invitrogen) using Lipofectamine 2000 according to manufacturer's instructions. After a 48-hr culture, knockdown efficiency was measured at the protein level by immunoblot analysis. Nonspecific siRNA (oligoID 12935-300: Invitrogen) was used as the negative control.

### Western blot analysis

Primary neuronal cells and SK-N-SH cells were lysed in lysis buffer [25 mM HEPES (4-(2-hydroxyethyl)-1-piperazineethanesulfonic acid), pH 7.4, 100 mM NaCl, 1 mM EDTA (ethylene diamine tetra acetic acid), 5 mM MgCl_2_, 0.1 mM DTT (dithiothreitol), and a protease inhibitor mixture]. Whole cell proteins were electrophoretically resolved on a 10%-15% sodium dodecyl sulfate polyacrylamide gel and transferred to a nitrocellulose membrane. Immunoreactivity was detected through sequential incubation with primary antibodies, horseradish peroxidase-conjugated secondary antibodies, and enhanced chemiluminescence reagents *i.e.* west save gold detection kit (AbFrontier Inc.). The primary antibodies used for immunoblotting were anti-LC3B (#4108, Cell Signaling Technology), anti-P62 (#5114, Cell Signaling Technology), anti-ATG5 (#2630, Cell Signaling Technology), anti-prion protein (ab52604, Abcam, Cambridge, MA, USA) and anti-β-actin (A5441, Sigma Aldrich). Images were examined using a Fusion FX7 imaging system (Vilber Lourmat, Torcy Z.I. Sud, France). Densitometry of the signal bands was analyzed using the Bio-1D software (Vilber Lourmat, Marne La Vallee, France).

### TEM (transmission electron microscopy) analysis

TEM samples were analyzed by Transmission Electron Microscope (JEM-2010, JEOL) installed in the Center for University-Wide Research Facilities (CURF) at Chonbuk National University. After fixation of SK-N-SH cell samples in 2.5% glutaraldehyde(TED PELLA, USA) in PBS (pH, 7.2), specimens were post fixed in 1% osmium tetroxide(Heraeus, South Africa), dehydrated in graded ethanol and propylene oxide(Acros Organics, USA), and then embedded in Epoxy resin(Embed812. NMA; Nadic methyl anhydride. DDSA; Dodenyl Succinic Anhidride. DMP-30., USA) as used previously. Serial ultrathin sections were cut on an LKB-III ultratome (LEICA, Germany). Ultrathin sections were stained with uranyl acetate (TED PELLA, USA) and lead citrate (TED PELLA, USA) and examined with the aid of a Hitachi H7600 electron microscope (Hitachi, Japan) at an accelerating voltage of 100 kV.

### Statistical analysis

The unpaired *t*-test or Welch's correction was used for comparison between the 2 groups. The one-way ANOVA followed by the Dunnett's test was used for multiple comparison. All statistical analyses were performed with GraphPad Prism software. Results were considered significant for values * *p* < 0.05, ** *p* < 0.01 or *** *p* < 0.001.

## References

[1] Costanzo M, Zurzolo C (2013). The cell biology of prion-like spread of protein aggregates: mechanisms and implication in neurodegeneration. The Biochemical journal.

[2] Sakudo A, Ano Y, Onodera T, Nitta K, Shintani H, Ikuta K, Tanaka Y (2011). Fundamentals of prions and their inactivation (review). International journal of molecular medicine.

[3] Prusiner SB Prions.

[4] Pan KM, Baldwin M, Nguyen J, Gasset M, Serban A, Groth D, Mehlhorn I, Huang Z, Fletterick RJ, Cohen FE Conversion of alpha-helices into beta-sheets features in the formation of the scrapie prion proteins.

[5] Oesch B, Westaway D, Walchli M, McKinley MP, Kent SB, Aebersold R, Barry RA, Tempst P, Teplow DB, Hood LE (1985). A cellular gene encodes scrapie PrP 27-30 protein. Cell.

[6] Brandner S, Isenmann S, Raeber A, Fischer M, Sailer A, Kobayashi Y, Marino S, Weissmann C, Aguzzi A (1996). Normal host prion protein necessary for scrapie-induced neurotoxicity. Nature.

[7] Forloni G, Angeretti N, Chiesa R, Monzani E, Salmona M, Bugiani O, Tagliavini F (1993). Neurotoxicity of a prion protein fragment. Nature.

[8] Chiesa R, Harris DA (2001). Prion diseases: what is the neurotoxic molecule?. Neurobiology of disease.

[9] Bueler H, Aguzzi A, Sailer A, Greiner RA, Autenried P, Aguet M, Weissmann C (1993). Mice devoid of PrP are resistant to scrapie. Cell.

[10] Prusiner SB, Groth D, Serban A, Koehler R, Foster D, Torchia M, Burton D, Yang SL, DeArmond SJ Ablation of the prion protein (PrP) gene in mice prevents scrapie and facilitates production of anti-PrP antibodies.

[11] Manson JC, Clarke AR, Hooper ML, Aitchison L, McConnell I, Hope J (1994). 129/Ola mice carrying a null mutation in PrP that abolishes mRNA production are developmentally normal. Molecular neurobiology.

[12] Giese A, Brown DR, Groschup MH, Feldmann C, Haist I, Kretzschmar HA (1998). Role of microglia in neuronal cell death in prion disease. Brain pathology (Zurich, Switzerland).

[13] Boellaard JW, Kao M, Schlote W, Diringer H (1991). Neuronal autophagy in experimental scrapie. Acta neuropathologica.

[14] Sikorska B, Liberski PP, Brown P (2007). Neuronal autophagy and aggresomes constitute a consistent part of neurodegeneration in experimental scrapie. Folia neuropathologica / Association of Polish Neuropathologists and Medical Research Centre, Polish Academy of Sciences.

[15] Cuervo AM (2004). Autophagy: in sickness and in health. Trends in cell biology.

[16] Bromme HJ, Holtz J (1996). Apoptosis in the heart: when and why? Molecular and cellular biochemistry.

[17] Cuervo AM (2004). Autophagy: many paths to the same end. Molecular and cellular biochemistry.

[18] Glick D, Barth S, Macleod KF (2010). Autophagy: cellular and molecular mechanisms. The Journal of pathology.

[19] Goold R, McKinnon C, Rabbanian S, Collinge J, Schiavo G, Tabrizi SJ (2013). Alternative fates of newly formed PrPSc upon prion conversion on the plasma membrane. Journal of cell science.

[20] Heiseke A, Aguib Y, Schatzl HM (2010). Autophagy, prion infection and their mutual interactions. Current issues in molecular biology.

[21] Yao H, Zhao D, Khan SH, Yang L (2013). Role of autophagy in prion protein-induced neurodegenerative diseases. Acta biochimica et biophysica Sinica.

[22] Xu Y, Tian C, Wang SB, Xie WL, Guo Y, Zhang J, Shi Q, Chen C, Dong XP (2012). Activation of the macroautophagic system in scrapie-infected experimental animals and human genetic prion diseases. Autophagy.

[23] Bjorkoy G, Lamark T, Brech A, Outzen H, Perander M, Overvatn A, Stenmark H, Johansen T (2005). p62/SQSTM1 forms protein aggregates degraded by autophagy and has a protective effect on huntingtin-induced cell death. The Journal of cell biology.

[24] Rubinsztein DC, Cuervo AM, Ravikumar B, Sarkar S, Korolchuk V, Kaushik S, Klionsky DJ (2009). In search of an “autophagomometer”. Autophagy.

[25] Oh JM, Shin HY, Park SJ, Kim BH, Choi JK, Choi EK, Carp RI, Kim YS (2008). The involvement of cellular prion protein in the autophagy pathway in neuronal cells. Molecular and cellular neurosciences.

[26] Barbieri G, Palumbo S, Gabrusiewicz K, Azzalin A, Marchesi N, Spedito A, Biggiogera M, Sbalchiero E, Mazzini G, Miracco C, Pirtoli L, Kaminska B, Comincini S (2011). Silencing of cellular prion protein (PrPC) expression by DNA-antisense oligonucleotides induces autophagy-dependent cell death in glioma cells. Autophagy.

[27] Pattingre S, Tassa A, Qu X, Garuti R, Liang XH, Mizushima N, Packer M, Schneider MD, Levine B (2005). Bcl-2 antiapoptotic proteins inhibit Beclin 1-dependent autophagy. Cell.

[28] Yang Z, Klionsky DJ (2009). An overview of the molecular mechanism of autophagy. Current topics in microbiology and immunology.

[29] Jeong JK, Park SY (2015). Melatonin regulates the autophagic flux *via* activation of alpha-7 nicotinic acetylcholine receptors. Journal of pineal research.

[30] Damme M, Suntio T, Saftig P, Eskelinen EL (2015). Autophagy in neuronal cells: general principles and physiological and pathological functions. Acta neuropathologica.

[31] Lee JH, Jeong JK, Park SY (2014). Sulforaphane-induced autophagy flux prevents prion protein-mediated neurotoxicity through AMPK pathway. Neuroscience.

[32] Ciechanover A, Kwon YT (2015). Degradation of misfolded proteins in neurodegenerative diseases: therapeutic targets and strategies. Experimental & molecular medicine.

[33] Nakagaki T, Satoh K, Ishibashi D, Fuse T, Sano K, Kamatari YO, Kuwata K, Shigematsu K, Iwamaru Y, Takenouchi T, Kitani H, Nishida N, Atarashi R (2013). FK506 reduces abnormal prion protein through the activation of autolysosomal degradation and prolongs survival in prion-infected mice. Autophagy.

[34] Czyzyk-Krzeska MF, Meller J, Plas DR (2012). Not all autophagy is equal. Autophagy.

[35] Mizushima N, Yoshimori T (2007). How to interpret LC3 immunoblotting. Autophagy.

[36] Lee JH, Moon JH, Kim SW, Jeong JK, Nazim UM, Lee YJ, Seol JW, Park SY (2015). EGCG-mediated autophagy flux has a neuroprotection effect *via* a class III histone deacetylase in primary neuron cells. Oncotarget.

[37] Jeong JK, Moon MH, Bae BC, Lee YJ, Seol JW, Kang HS, Kim JS, Kang SJ, Park SY (2012). Autophagy induced by resveratrol prevents human prion protein-mediated neurotoxicity. Neuroscience research.

[38] Kaufman SK, Diamond MI (2013). Prion-like propagation of protein aggregation and related therapeutic strategies. Neurotherapeutics.

[39] Aguzzi A, Calella AM (2009). Prions: protein aggregation and infectious diseases. Physiological reviews.

[40] Nah J, Pyo JO, Jung S, Yoo SM, Kam TI, Chang J, Han J, Soo AAS, Onodera T, Jung YK (2013). BECN1/Beclin 1 is recruited into lipid rafts by prion to activate autophagy in response to amyloid beta 42. Autophagy.

[41] Shin HY, Park JH, Carp RI, Choi EK, Kim YS (2014). Deficiency of prion protein induces impaired autophagic flux in neurons. Frontiers in aging neuroscience.

[42] Oh JM, Choi EK, Carp RI, Kim YS (2012). Oxidative stress impairs autophagic flux in prion protein-deficient hippocampal cells. Autophagy.

[43] Ettaiche M, Pichot R, Vincent JP, Chabry J (2000). *In vivo* cytotoxicity of the prion protein fragment 106-126. The Journal of biological chemistry.

